# Eating disorders with over-exercise: A cross-sectional analysis of the mediational role of problematic usage of the internet in young people

**DOI:** 10.1016/j.jpsychires.2020.11.004

**Published:** 2020-11-04

**Authors:** Konstantinos Ioannidis, Roxanne W Hook, Jon E Grant, Katarzyna Czabanowska, Andres Roman-Urrestarazu, Samuel R Chamberlain

**Affiliations:** a Department of Psychiatry, University of Cambridge, UK; b Cambridge and Peterborough NHS Foundation Trust, Cambridge, UK; c Care and Public Health Research Institute, Maastricht University, Maastricht, Netherlands; d Department of Psychiatry and Behavioral Neuroscience, University of Chicago, Chicago, Illinois, USA; e Institute of Public Health, Faculty of Health Sciences, Jagiellonian University, Krakow, Poland

**Keywords:** eating disorder, anorexia nervosa, bulimia nervosa, internet addiction, problematic internet use

## Abstract

**Background:**

Eating disorders are widespread illnesses with significant impact. There is growing concern about how young people overuse online resources leading to mental health sequelae.

**Methods:**

We gathered data from 639 individuals from a population cohort. Participants were all young adults at the point of contact and were grouped as having probable eating disorder with excessive exercise (n=37) or controls (n=602). We measured obsessionality, compulsivity, impulsivity, and problematic internet use. Group differences in these domains were evaluated; and structural equation modelling (SEM) was used to assess structural relationships between variables.

**Results:**

Cases had higher scores of obsessional thoughts of threat (Cohen’s d=0.94, p <0.001), intolerance towards uncertainty (Cohen’s d=0.72; p <0.001), thoughts of importance and control (Cohen’s d=0.65, p <0.01), compulsivity (Cohen’s d=0.72; p <0.001), negative urgency (Cohen’s d=0.75, p<0.001), and higher problematic usage of the internet (Cohen’s d=0.73; p-corrected <0.001). Our SEM showed significant partial mediation of problematic internet use on both the effect of obsessionality latent factor on cases (z-value=2.52, p<0.05), as well as of sensation seeking latent factor on cases (z-value=2.09, p<0.05).

**Discussion:**

Youth with eating disorder and heightened exercise levels have increased obsessive thoughts of threat, compulsivity traits and sensation seeking impulsivity. The association between obsessive thoughts and eating disorders, as well as sensation seeking and eating disorder symptoms were partially mediated by problematic internet use. Excessive use of online resources may be playing a role in the development or maintenance of eating disorder symptoms in the background of obsessional thoughts and sensation seeking impulsive traits.

## Introduction

Eating disorders (EDs) have the highest morbidity and mortality of all mental illnesses (Arcelus, Mitchell, Wales, & Nielsen, 2011) and affect a significant proportion of the population. Depending on the cohort and definition, anorexia nervosa (AN) has a lifetime prevalence of between 1.2% and 4.3% (broad definition) in females (Smink, Van Hoeken, & Hoek, 2012) and 0.24% in men, whereas bulimia nervosa (BN) has a lifetime prevalence of 1.0-2.9% in females, and 0.5% in men (Smink et al., 2012). Incidence of AN has increased 50-fold since the 1930s and has remained relatively stable since the 1970s (Hoek, 2006); however, some studies suggest an ongoing increase of incidence in younger populations (Zipfel, Giel, Bulik, Hay, & Schmidt, 2015) and eating disorders still remain an important health burden for societies worldwide (Erskine, Whiteford, & Pike, 2016; Treasure et al., 2015).

### Problematic usage of the internet and eating disorders

Over the last decade, there has been growing concern over the impact of online media on eating disorders (Ioannidis et al., 2020; Mingoia, Hutchinson, Wilson, & Gleaves, 2017; Tiggemann & Miller, 2010; Tiggemann & Slater, 2013, 2017). Problematic usage of the internet (PUI) is an umbrella term used to describe maladaptive behaviors manifesting on the online milieu (Fineberg et al., 2018) and PUI is an age-related multifaceted construct that encompasses a number of maladaptive online behaviors (Ioannidis et al., 2018) linked with heightened levels of psychiatric comorbidity (Ho et al., 2014). Cross-sectional correlations between PUI and eating disorder psychopathology, body dissatisfaction, restrained eating, drive for thinness have been shown in meta-analysis (Ioannidis et al., 2020), while studies of bulimia and PUI show similar correlations and group comparisons (Butkowski, Dixon, & Weeks, 2019; Melioli, Rodgers, Rodrigues, & Chabrol, 2015; Smith, Hames, & Joiner, 2013; Tao, 2013). A number of prospective studies support the notion that effects of PUI on eating disorders do exist and exposure to particular types of online content e.g. social networking site (SNS) use may have accumulating effects over time (de Vries, Peter, de Graaf, & Nikken, 2016; Ferguson, Muñoz, Garza, & Galindo, 2014; Hsieh, Hsiao, Yang, Liu, & Yen, 2018; Hummel & Smith, 2015; Smith et al., 2013; Tiggemann & Slater, 2017). Experimental studies in the field have demonstrated direct effects of SNS usage or consumption of pro-ED content (e.g. “fitspiration”) on body dissatisfaction, internalization of the thin ideal and weight and shape concerns (Fardouly, Diedrichs, Vartanian, & Halliwell, 2015; Mabe, Forney, & Keel, 2014; Prichard, Kavanagh, Mulgrew, Lim, & Tiggemann, 2020; Slater, Halliwell, Jarman, & Gaskin, 2017; Tiggemann & Zaccardo, 2015).

### Excessive exercise

Excessive exercise is a particularly challenging eating disorder behavior that can lead to catastrophic consequences e.g. precipitous weight loss, coupled with exercise through injury, heart abnormalities (e.g. life threatening bradycardia), rhabdomyolysis, among other complications (Ghoch, Calugi, & Grave, 2016; Peñas-Lledó, Vaz Leal, & Waller, 2002). AN cohorts present with excessive exercise in up to ~80% (Rizk, Lalanne, Berthoz, Kern, & Godart, 2015)and in a 15-year-prospective study of AN showed compulsive excessive exercise at the time of discharge being one of the most significant predictors of chronic outcome and early time to relapse (HR = 2.2, 95% CI = 1.1–4.9) (Strober, Freeman, & Morrell, 1997). Excessive exercise has been linked with the consumption of “thinspiration” or “fitspiration” online content (Carrotte, Vella, & Lim, 2015; Quesnel, Cook, Murray, & Zamudio, 2018), as well as weight loss and fitness applications (Apps) in both males and females (Almenara, Machackova, & Smahel, 2019; Embacher Martin, McGloin, & Atkin, 2018; Levinson, Fewell, & Brosof, 2017; Linardon & Messer, 2019; Simpson & Mazzeo, 2017).

### Obsessionality, intolerance of uncertainty and compulsivity in eating disorders

Obsessional ideas about own body image are core symptoms of AN (Collier & Treasure, 2004) and have causally been linked to starvation since the early exploration of consequences of starvation to healthy individuals (Minnesota study) (Keys, Brozek, Henschel, Mickelsen, & Taylor, 1950). In the 1980s’ EDs were considered as extreme manifestations of societal obsession with thinness (Collier & Treasure, 2004) and we now know that AN has a genetic linkage with obsessionality on chromosome 1 locus (Devlin, 2002). Furthermore, restricting AN was demonstrated to have reduced cognitive flexibility both during AN episodes and after recovery (Tchanturia et al., 2004), while individuals with AN experience obsessional thoughts linked to their compulsive exercise, eating and weight related obsessionality (Byrne et al., 2018; Godier & Park, 2015).

Linked to obsessional traits, ‘intolerance of uncertainty’ (IU) is the tendency for a negative emotional, cognitive and behavioral reaction to uncertain situations and events. Compulsive eating disorder behaviors have been linked to IU (Boswell, Thompson-Hollands, Farchione, & Barlow, 2013; Parkes et al., 2019). IU has been quantitatively demonstrated as prevalent in AN (Frank et al., 2012; Sternheim, Startup, & Schmidt, 2011) and qualitatively explored to show that IU in AN manifests as fear of unduly evaluation from others, leading to social problem solving difficulties (Sternheim, Danner, van Elburg, & Harrison, 2020) and compulsive planning and action (Sternheim, Konstantellou, Startup, & Schmidt, 2011).

Compulsivity has been defined as a trait in which actions are persistently repeated despite adverse consequences (Robbins, Gillan, Smith, de Wit, & Ersche, 2012). Behavioral traits of compulsivity covary with eating disorder psychopathology (Godier & Park, 2014). Extreme dietary restriction and over-exercise may reflect excessive habit formation leading to compulsive starvation or over-activity behavior (Everitt & Robbins, 2005; Fladung et al., 2010).

### Impulsivity and sensation seeking in eating disorders

Impulsivity is a multi-faceted construct referring to acting without forethought or reflection or consideration of the consequences. PUI has been linked with increased levels of trait impulsivity and compulsivity (Ioannidis et al., 2016) and sensation seeking (Lin & Tsai, 2002). Impulsivity in eating disorders has been linked to poor long-term AN outcomes (Fichter, Quadflieg, & Hedlund, 2006), but also strongly related with bulimia with or without purging and binge eating disorder (Collier & Treasure, 2004; Fahy & Eisler, 1993). Heightened sensation seeking impulsivity has been particularly demonstrated in bulimia as compared to controls (Rossier, Bolognini, Plancherel, & Halfon, 2000) even after controlling for victimization and traumatic experiences (Brewerton, Cotton, & Kilpatrick, 2018). Impulsivity, compulsivity and obsessionality, when considered together they are found as prevalent behaviors in purging anorexia (Hoffman et al., 2012). Obsessional thinking is strongly positively correlated with compulsive behavior(Kim et al., 2016). Impulsivity and compulsivity exist cross-diagnostically in latent functionally impairing forms which are positively correlated (Chamberlain, Stochl, Redden, & Grant, 2018).

### Aims and hypotheses

This current study had two aims: first we aimed to compare the behavioral characteristics of eating disorder traits with heightened levels of exercise in respect to their levels of (1) impulsivity, (2) compulsivity, (3) obsessionality, (4) sensation seeking, (5) intolerance to uncertainty and (6) problematic usage of the internet against controls. By doing so, we aim to quantify differences on group level in our dataset, as they have been demonstrated in previous research, and establish that our cohort does share the behavioral characteristics in line with current literature. Therefore, we hypothesized that participants with eating disorders and heightened exercise will present with increased levels of trait impulsivity, compulsivity, obsessionality, as well as heightened levels of intolerance for uncertainty and sensation seeking when compared to controls. Our second aim would be to statistically explore the structural relationship between the variables in our model and consecutively the potential mediating effect that problematic internet use may have on these neurobiological dimensions on their effect on eating disorders. To date there is no study exploring those mediating effects of PUI in eating disorders.

## Methods

### Study criteria and recruitment

Participants were recruited from the Neuroscience in Psychiatry Network (NSPN) UK youth cohort:, which is a longitudinal cohort, exploring brain development trajectories and mental health outcomes (Kiddle et al., 2018). The sample was originally recruited on an age-sex stratified basis, in order to maximize representativeness of the normal population in the catchment areas covered (Cambridge and London). In this study, we contacted all individuals (adults, Mean [sd] age: 23.4 [3.2]) who were still enrolled in this cohort at the time of data collection (2017–2018) via email and invited them to take part in an online study being conducted via SurveyMonkey. Participants received £15 compensation in the form of a gift voucher. Further methodological details about the recruitment and instruments are presented in previous work (Chamberlain et al., 2019). The data that support the findings of this study are available on request from the corresponding author, subject to agreement of the Chief Investigator. The data are not publicly available due to privacy or ethical restrictions

### Ethical considerations

The procedures of this study were carried out in accordance with the Declaration of Helsinki and the study was approved by the Cambridge East Research Ethics Committee (Study approval number 16/EE/0260). All subjects gave informed consent online.

### Assessments

Participants were classified as cases according to whether or not they displayed high probability of having an eating disorder diagnosis and excessive exercise. For the assessment of their eating disorder diagnosis we used the SCOFF Eating Disorder Questionnaire (Luck et al., 2002). This is a validated screening tool for detection of eating disorders, specifically either anorexia nervosa or bulimia nervosa. The scale is sensitive to the presence of different aspects of eating disorder symptoms, such as purging, weight loss, distorted body image, loss of control over eating and food preoccupation. A score of 2 and above indicates high likelihood of an eating disorder reason why we used this cut-off to define our cases. For the assessment of excessive exercise, we used the exercise addiction inventory (EAI) (Griffiths, Szabo, & Terry, 2005). Due to prior absence of an established cut-off, we defined excessive exercise based on scores being >1 standard deviation from that of the cohort that was examined (19 and above; the mean cohort score was 13.4). Therefore, our cases were characterized as probable eating disorder (either anorexia nervosa or bulimia nervosa) plus excessive exercise symptoms.

### Behavioral Assessments

#### Problematic usage of the internet

***Problematic usage of the internet*** was quantified using the Internet Addiction Test short-12 item version (IAT-12) (Pawlikowski, Altstötter-Gleich, & Brand, 2013). The IAT-12 consists of twelve items ascertaining the level of problematic internet use, and was developed from Kimberley Young’s Internet Addiction Test, based on rigorous psychometric refinement of the original scale.

#### Compulsivity

***Cambridge–Chicago Compulsivity Trait Scale*** (CHI-T) (Chamberlain & Grant, 2018). This is a scale designed to capture the comprehensive aspects of compulsivity, viewed trans-diagnostically. The scale comprises 15 items, each scored on a Likert scale of 1–4, from “strongly disagree” to “strongly agree.” The total score is 60, with higher scores indicating higher compulsivity. The scale is sensitive to compulsivity across a range of pathologies, such as disordered gambling, substance use, and obsessive-compulsive symptoms (Albertella et al., 2019).

#### Impulsivity

***The Barratt Impulsiveness Scale*** short version (BIS-8) to quantify impulsive personality (Patton, Stanford, & Barratt, 1995) is a self-report questionnaire used to determine levels of impulsiveness.

***The short Urgency, Premeditation (lack of), Perseverance (lack of), Sensation Seeking, Positive Urgency, Impulsive Behavior Scale*** (S-UPPS) is a measurement of impulsivity as a multi-faceted and multi-dimensional construct, comprising five impulsive personality traits (Whiteside & Lynam, 2001). The proposed model of impulsivity includes five specific (distinct) dispositions a) sensation seeking (i.e. pursuit of novel/exciting stimuli), b) lack of planning (i.e. action without advanced planning), c) lack of perseverance (i.e., limited capacity for focus maintenance), d) positive urgency (i.e., rash action in response to intense positive emotions), and e) negative urgency (i.e., rash action in response to intense negative emotions) (Whiteside & Lynam, 2001). The S-UPPS scale has five subscales (first order factors) and four items per sub-scale. Sensation seeking comprises a second order factor alone, whilst positive and negative urgency comprise ‘emotion based rushed action’ and lack of premeditation and perseverance comprise ‘deficits in conscientiousness’ (Cyders, Littlefield, Coffey, & Karyadi, 2014).

#### Sensation Seeking

***The Brief Sensation Seeking Scale*** (BSSS-8) is a measure of sensation seeking as a psychobiological trait of need for novelty, complexity and intensity (Hoyle, Stephenson, Palmgreen, Lorch, & Donohew, 2002). It comprises of eight items of a five-point-Likert scale from “strongly disagree” to “strongly agree”.

#### Intolerance of Uncertainty

***Intolerance of Uncertainty scale*** (Buhr & Dugas, 2002). This is a scale original developed in French, but validated for English speakers as well. The scale comprises 27 items, each scored on a Likert scale of 1–5, from “not at all characteristic of me” to “entirely characteristic of me”; with higher scores indicating higher degree of intolerance to uncertainty. The measure of interest was the total score.

#### Statistical analysis

**Figure 1:**
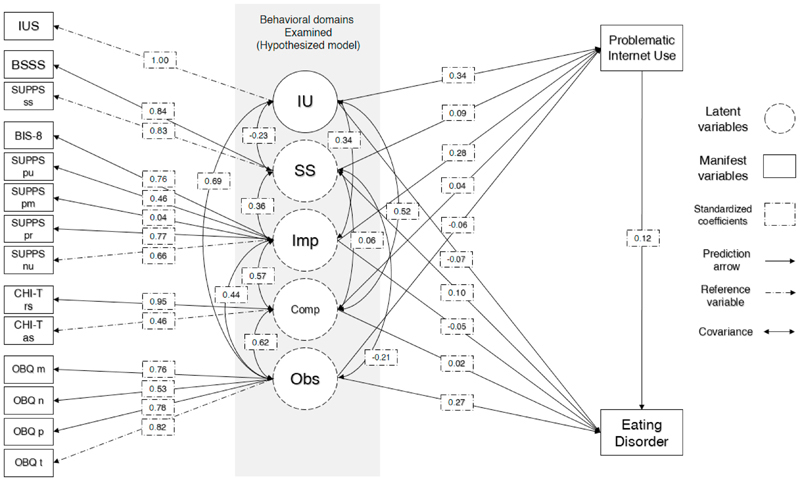
Structural equation model hypothesized model **Legend: IUS** = intolerance to uncertainty score; **IU** = intolerance to uncertainty, as directly measured by IUS; **SS** = sensation seeking latent factor; **BSSS** = Brief Sensation Seeking Scale (BSSS) total score; **BIS-8** = The Barratt Impulsiveness Scale, short version total score; **Imp** = impulsivity latent factor; **SUPPS** = Short UrgencyPremeditation-Perseverance-Sensation Seeking-Positive Urgency Scale (SUPPS); **SUPPS pu** = SUPPS Positive urgency; **SUPPS pr** = SUPPS (Lack of) perseverance; **SUPPS pm** = SUPPS (Lack of) premeditation; **SUPPS nu** = SUPPS Negative urgency; **CHI-T** = Cambridge-Chicago Compulsivity Trait Scale (CHI-T); **CHI-T rs** = CHI-T reward-seeking and need for perfection; **CHI-T as** = CHI-T anxiolytic/soothing compulsivity; **Comp** = compulsivity latent factor; **Obs** = obsessionality latent factor; **OBQ** = Obsessive Beliefs Questionnaire, short version (OBQ-20); **OBQ m** = Obsessional thoughts of importance and control; **OBQ n** = Obsessional intolerance towards uncertainty; **OBQ p** = Obsessional thoughts of inflated responsibility; **OBQ t** = Obsessional thoughts of threat; **Problematic Internet use** = Internet Addiction Test, short version (IAT-12) total score; **Eating disorder** = case; Numeric scores are standardized regression coefficients in direct lines and standardized covariance coefficients in curves lines.

Data processing and statistical analyses were conducted using statistical software R version 3.4.2 and “dplyr” (Wickham, François, Henry, Müller, & RStudio, 2020) and “lavaan” (Rosseel, 2012) R packages. We performed direct comparisons of our cases and controls using student t-test under the assumption of normal distribution of behavioral characteristics in our cohort. The NSPN is a representative cohort of the catchment area and behavioral characteristics are expected to have normal distributions. We used chi-square to compare non-parametric values e.g. gender. Finally, we also performed a structural equation modelling (SEM) to explore structural relationships between the variables at hand; this also enabled us to ascertain whether problematic internet use has any mediation influence on the effect of behavioral traits on eating disorder cases. Our SEM initial (hypothesized) model included four latent variables predicted by manifest variables as such: a) ‘Obsessionality’ latent factor predicted by the four subscales of OBQ (“Obsessional thoughts of threat”, “Obsessional intolerance towards uncertainty”, “Obsessional thoughts of importance and control”, “Obsessional thoughts of inflated responsibility”; b) “Compulsivity” latent factor predicted by the two factors of CHI-T “reward-seeking and need for perfection” and “anxiolytic/soothing compulsivity”; c) “Impulsivity” latent factor predicted by the four factors of S-UPPS “Negative urgency”, “Lack of perseverance”, “Lack of premeditation”, “Positive urgency”, also “BIS-8 total score”; d) “Sensation seeking” latent factor predicted by “BSSS total score” and “S-UPPS Sensation seeking”. We hypothesized that those variables were predictive of problematic usage of the internet “IAT-12 total score”, as well as “cases”, as defined above (SCOFF≥2, EAI≥19). We also used “Intolerance to Uncertainty” as (IUS total score) as a separate path, predicting both PUI and “cases”. We calculated regression coefficients for all predictors as well as covariances between all latent variables between themselves and with IUS. We calculated the indirect effect of problematic internet use for every latent and IUS variable on cases. Our initial (hypothesized) model was plotted and presented in Figure 1.

We then followed a step wise change of our model by adding relationships based on their modification indices and subtracting relationships based on non-significant covariances, aiming to improve the model’s goodness of fit statistics. We added new relationships with high modification indices taking into account the theoretical implications of adding those relationships into the model. We calculated the degrees of freedom, goodness and badness of fit statistics (“AIC” = Akaike information criterion; “CFI” = comparative fit index; “TLI” = Tucker–Lewis index; “RMSEA” = root mean square error) for every model and compared each model with the previous one using chi-square comparisons. We finalized our SEM when reached non-significant improvement in our model via path change (Schermelleh-Engel, Moosbrugger, & Müller, 2003).

## Results

Our final sample comprised 37 cases (i.e. individuals meeting criteria for probable eating disorder plus having excessive exercise), and 602 controls. Group comparison results are shown in Table 1.

### Case control comparison

Cases had higher scores of obsessional thoughts of threat (t-test, Cohen’s d=0.94, p<0.001), obsessional intolerance towards uncertainty (OBQ) (Cohen’s d=0.72; p<0.001), obsessional thoughts of importance and control (Cohen’s d=0.65, p<0.01), transdiagnostic compulsivity traits (Cohen’s d=0.72; p<0.001), negative urgency (Cohen’s d=0.75, p<0.001), intolerance of uncertainty (IUS) (Cohen’s d=0.55; p <0.05), and higher problematic usage of the internet (Cohen’s d=0.73; p corrected <0.001). All other results were non-significant or became non-significant after the application of Bonferroni correction for multiple comparisons. We used standardized mean difference (Cohen’s d) under the assumption of normality and homogeneity of variances.

### Structural equation modelling

**Figure 2:**
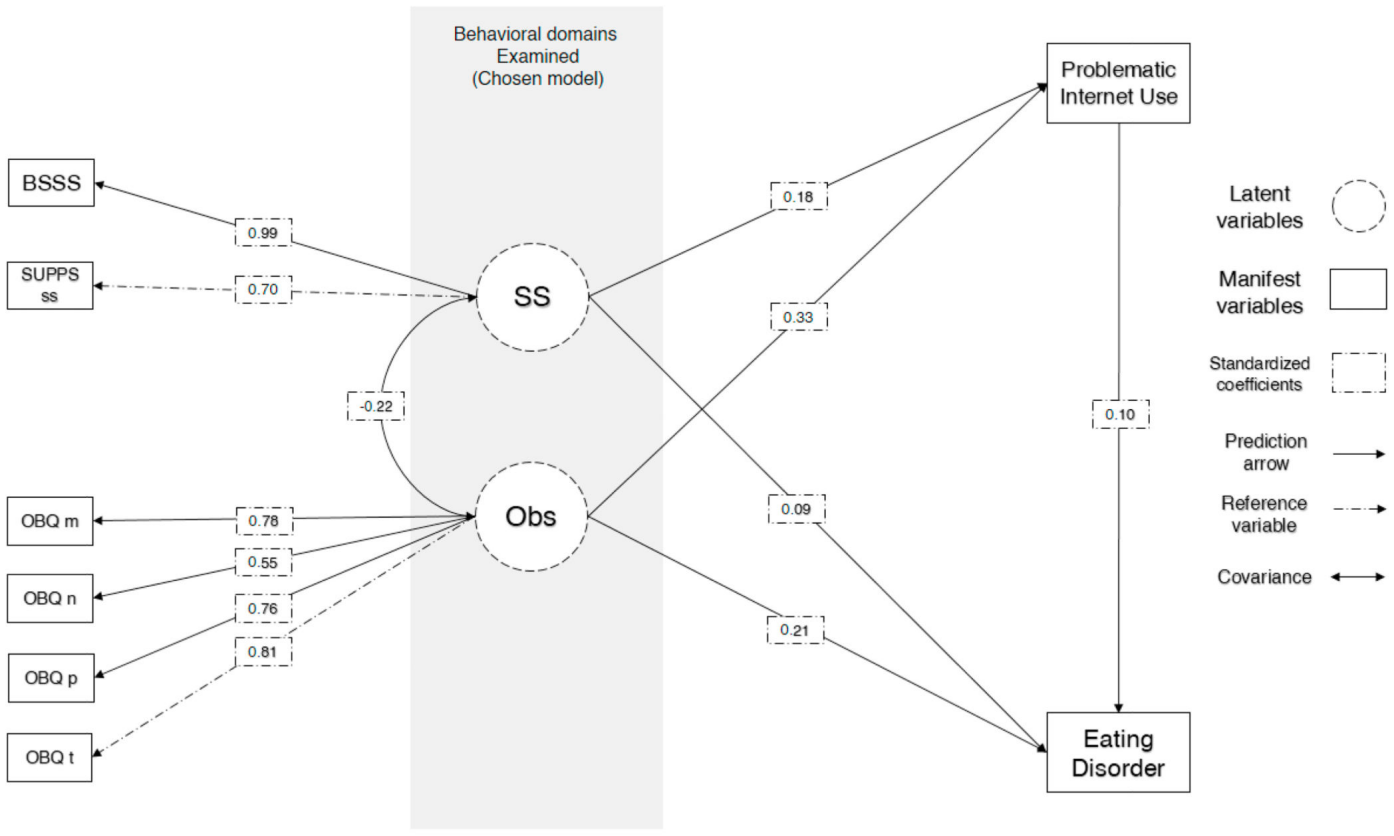
Structural equation model chosen model **Legend: BSSS** = Brief Sensation Seeking Scale (BSSS) total score; **BIS-8** = The Barratt Impulsiveness Scale, short version total score; **Imp** = impulsivity latent factor; **OBQ** = Obsessive Beliefs Questionnaire, short version (OBQ-20); **OBQ m** = Obsessional thoughts of importance and control; **OBQ n** = Obsessional intolerance towards uncertainty; **OBQ p** = Obsessional thoughts of inflated responsibility; **OBQ t** = Obsessional thoughts of threat; **Problematic Internet use** = Internet Addiction Test, short version (IAT-12) total score; **Eating disorder** = case; Numeric scores are standardized regression coefficients in direct lines and standardized covariance coefficients in curves lines.

Problematic internet use was associated with obsessionality (regression coef. z=7.61, p<0.001) and sensation seeking (z=4.65, p<0.001); eating disorder case was associated with obsessionality (z=4.53, p<0.001), sensation seeking (z=2.26, p<0.02) and PUI (z=2.32, p<0.02). The chosen model with the lowest RMSEA was model 8 (see Table 2) which had Comparative Fit Index (CFI) 0.975 and Tucker-Lewis Index (TLI) 0.956 indicating good fit. Root Mean Square Error of Approximation was less than 0.1 (mean=0.059; 1000-iterations-bootstrap 95%CI 0.059-0.060). The indirect (mediation) effect of PUI on obsessionality effect on cases was statistically significant (z=2.25, p=0.024) indicating partial mediation (Obsessionality ~ case standardized effect reduction from 0.24 to 0.21 [12.5% reduction]). The indirect (mediation) effect of PUI on sensation seeking effect on cases was statistically significant (z=2.08, p=0.037) indicating partial mediation (Sensation seeking ~ case standardized effect reduction from 0.11 to 0.09 [18% reduction]). Initial models did not have acceptable goodness of fit statistics and were rejected (see Table 2). Full mediation SEM results are presented in Table 3. Model 8 (chosen model) is graphically presented in Figure 2. Comparative statistics between hypothesized model and final model are presented in Table 2.

## Discussion

This is the first study to examine the problematic online behaviors, coupled with behavioral characteristics of a putative eating disorders cohort with heightened excessive exercise behaviors. In our study, we identified, through group comparisons, that cases, as compared to controls, had heightened degree of obsessive thoughts of threat, obsessional intolerance towards uncertainty, obsessional thoughts of importance and control, high cross-diagnostic traits of compulsivity, negative urgency impulsivity and higher levels of problematic internet use. Those results are in line with previous research, that obsessional preoccupation with food and food predominance, as well as and intolerance to uncertainty manifesting with deficits in social decision making, planning and action, as well as fear of unduly evaluation from others (Davis & Kaptein, 2006; Sternheim, Konstantellou, et al., 2011). Increased compulsivity, manifesting as compulsive restriction of food intake and compulsive exercise is also in line with previous research (Davis & Kaptein, 2006; Godier & Park, 2014), as well as a higher level of negative urgency impulsive, particularly in cohorts of heightened impulsivity during negative emotional states (e.g. binge/purging AN or BN) (Westwater et al., 2019). The increased level of PUI is also in line with previous research.

Furthermore, our study is the first to explore the mediation effect of problematic internet behaviors on the impact of obsessionality and sensation seeking, to eating disorder symptoms, via SEM. Our analysis showed partial mediation, for both effects of obsessionality (0.24 to 0.21) and sensation seeking (0.11 to 0.09) suggestive that obsessional thoughts and sensation seeking impulsivity traits, when present in in young populations, may be impacting on the development or perseverance of eating disorders, partially via the problematic usage of online resources. While a SEM analysis does not provide evidence for causal directional link, it highlights the importance for future studies that can potentially examine this interaction further. Previous longitudinal research has shown that the use of social media (Facebook) maintained weight and shape concerns as well as state anxiety (Mabe et al., 2014) as compared to alternate online activity. Also, social media use has been associated with perseverance of obsessional body image symptoms (Tiggemann & Slater, 2013) and found to causally associate with obsessive drive for thinness longitudinally (Tiggemann & Slater, 2017). We argue that our mediation model is grounded on robust theory of obsessional thoughts and sensation seeking behavior strongly associate with both with PIU and ED and the mediation pathway in proposition is both statistically demonstrable and theoretically plausible. We argue that enhancing our understanding of the behavioral underpinnings of this effect may be helpful in the developing appropriate interventions and therapeutic targets, including health recommendations about the use of novel technology, digital interventions and appropriate clinical interviews and screening of symptoms. Obsessional thoughts linked with compulsive usage of the internet (e.g. calorie counting via apps, fitness apps, obsessing over body image content consumption, step counting etc.) and sensation seeking online behaviors (e.g. consumption of ‘fitspiration’ or food related or ‘ mukbang’ content etc.) may be potential such targets. Finally, the current manuscript is prepared in the unusual times of the COVID-19 pandemic. The global social distancing measures have driven people to rely more that even on online resources for their work, leisure and social connectedness. It is unclear what effect this may have, but it is possible that we may see higher levels of problematic usage of the internet in the future; this may mean that it would be pertinent for future research to unravel the causal links between behavioral traits predisposing for both PUI and EDs, to enable us to think about how to target those in our diagnostics, therapies and prevention programs.

### Limitations

We have several limitations to consider in this study deriving from our data collection process and instruments used. Given that this is an online survey, it has less quality control and less accuracy for measuring psychopathology constructs as compared to face-to-face clinical assessments. For example, we used the SCOFF questionnaire to ascertain putative AN or BN diagnosis. While the SCOFF is an efficient screening tool for AN and BN, and its specificity and positive predictive value are reasonable (Spec.: 89.6%, PPV: 24.4%) (Luck et al., 2002) for a screening tool, it does not have ‘gold-standard’ diagnostic validity that can be provided by a clinical or DSM-5 structured interview. Furthermore, due to the survey being delivered online, there is also a potential sampling bias, since returning participants of the NSPN cohort may be those who are more technologically adept or responsive to email requests. In respect to our SEM analysis, it is important to note that mediational models are presumptuously causal models in which the mediator is presumed to cause the outcome and not vice versa (Baron & Kenny, 1986). Here, we model on the basis that latent cross-diagnostic traits e.g. sensation seeking, compulsivity, obsessionality are factors predisposing to eating disorder behaviors, however, we cannot draw causal effects; this would require a different study design. Future research with appropriate (longitudinal, randomized, controlled) design can explore further whether those causal links exist and in which direction. Furthermore, for our SEM we used the CHI-T two-factor structure as reported in first publication (Chamberlain & Grant, 2018), however this factor analysis is considered preliminary; future research on the instrument in larger samples may replicate this finding or demonstrate a different factor structure for the instrument. It is important to note that BSSS total score and sensation seeking UPPS scores considered individually were not statistically significantly higher for cases in group comparisons; however, the sensation seeking latent factor was predictive of cases (p=0.02, see Table 3). This may imply that latent sensation seeking as modelled in our SEM brings together a wider range of sensation seeking parameters, rendering the latent construct predictive of eating disorder symptoms, an attribute that the instruments may not possess if considered individually.

### Conclusion

We have shown that our case group of putative eating disorders with heightened levels of activity have increased levels of obsessionality, cross-diagnostic compulsivity, negative urgency impulsivity, intolerance of uncertainty and higher levels of problematic usage of the internet. We have demonstrated that obsessionality latent factor and sensation seeking latent factor predict cases, and that problematic usage internet resources mediates that relationship. This mediation provides us novel insight into the potential role of problematic use of online resources for the development and perseverance of eating disorder psychopathology with heightened exercise levels.

## Figures and Tables

**Table 1:** Demographic and behavioral characteristics of study cohort

	TOTAL N = 639	Controls N = 502	ED Low exercise N= 100	ED High exercise N= 37	Group t-test comparison	p-value†	Signif. ††	Cohen’s d
	Mean (sd)	Mean (sd)	Mean (sd)	Mean (sd)				
**Age**	23.4(3.2)	23.4(3.1)	23.4(3.9)	23.4(3.1)	-	-	-	-
**Gender [%Female]**	65%	64.7%		70%	A < B			
**Obsessional thoughts of threat (OBQ)**	15.7 (6.1)	14.8(5.8)	18.1 (6.4)	21.0 (5.0)	A < B < C			
**Obsessional intolerance towards uncertainty (OBQ)**	18.2 (6.9)	17.3 (6.6)	20.6 (7.5)	22.8 (6.2)	A < B , C			
**Obsessional thoughts of importance and control (OBQ)**	14.1 (6.2)	13.4 (5.9)	16.4 (6.7)	17.8 (6.0)	A < B , C			
**Obsessional thoughts of inflated responsibility (OBQ)**	20.9 (6.3)	20.7 (6.4)	21.4 (6.3)	22.6 (5.6)	-			
**Transdiagnostic compulsivity traits (CHI-T)**	24.3 (6.0)	23.6 (5.9)	26.2 (6.0)	28.3 (4.8)	A < B < C			
**Impulsivity traits (BIS-8)**	16.4 (3.9)	15.9 (3.7)	18.4 (4.3)	17.1 (3.6)	A < B			
**Negative urgency (SUPPS)**	4.74 (2.49)	4.3 (2.3)	6.4 (2.4)	6.5 (2.1)	A < B , C			
**Lack of perseverance (SUPPS)**	4.44 (1.85)	4.4 (1.8)	4.8 (2.1)	3.7 (1.6)	A, B > C			
**Lack of premeditation (SUPPS)**	3.95 (1.85)	3.8 (1.8)	4.7 (2.0)	3.9 (2.2)	A < B			
**Sensation seeking (SUPPS)**	5.99 (2.60)	6.1 (2.6)	5.4 (2.6)	6.6 (3.1)	A, C > B			
**Positive urgency (SUPPS)**	3.14 (2.14)	3.0 (2.1)	3.8 (2.2)	4.0 (2.1)	A < B, C			
**Intolerance of Uncertainty (IUS)**	58.3 (21.1)	55.3 (20.0)	69.2 (22.7)	69.0 (18.9)	A < B, C			
**BSSS**	24.11(6.8)	24.2 (6.6)	23 (7.1)	25.5 (7.0)	-			
**Internet use (IAT-12)**	13.1(8.0)	11.7 (7.2)	16.6 (9.9)	18.5 (7.7)	A < B, C			
**Quality of life**	55.3 (17.8)	57.3 (17.6)	46.5 (15.4)	50.9 (17.7)	A > B, C			

Group A = controls; Group B = ED with low exercise; Group C = ED with high exercise;

† Two sample t-test p-values;

†† Significance: ‘*’ <0.05; ‘**’ <0.01; ‘***’ <0.001;

††† Chi-square; ED = Eating disorders; Obsessive Beliefs Questionnaire, short version (OBQ-20); Internet Addiction Test, short version (IAT-12); Cambridge–Chicago Compulsivity Trait Scale (CHI-T); The Barratt Impulsiveness Scale, short version (BIS-8); Brief Sensation Seeking Scale (BSSS); Short Urgency-Premeditation-Perseverance-Sensation Seeking-Positive Urgency Scale (SUPPS).

**Table 2:** Structural equation modelling

Model	DF	*χ*^2^ diff	Pr (>Chisq)	AIC	CFI	TLI	RMSEA	95%CI RMSEA	Path
**FIRST MODEL**	90	-	-	44816.52	0.653	0.538	0.158	0.158 - 0.158	-
**2**	89	187.68	***	44624.60	0.706	0.603	0.146	0.146 - 0.147	comp_rsfr ~~ supps_lackpersevrnce
**3**	88	23.463	***	44611.65	0.712	0.607	0.146	0.145 - 0.146	comp_rsfr ~~ bis8_total
**4**	87	122.89	***	44488.21	0.748	0.653	0.137	0.137 - 0.137	bis8_total ~~ supps_lackpremed
**5**	67	147.12	***	39466.08	0.735	0.640	0.143	0.142 - 0.143	Compulsivity =~ comp_rsfr + comp_arss
**6**	66	63.713	***	39395.04	0.759	0.667	0.137	0.137 - 0.137	OBQ_perfec_intoluncert ~~ supps_lackpersevrnce
**7**	22	342.93	***	28183.77	0.792	0.659	0.164	0.164 - 0.164	Impulsivity =~ exogenous
**8**	16	284.52	***	23472.67	0.971	0.949	0.062	0.062 - 0.063	ius_total ~
**9**	8	11.157	0.19	17743.64	0.970	0.944	0.072	0.071 - 0.072	SS =~ bsss_total + supps_sensseek

**Legend:** Signif. codes: 0 ‘***’ 0.001 ‘**’ 0.01 ‘*’ 0.05 ‘.’ 0.1 ‘ ’ 1 ; **DF** = Degrees of freedom; ***χ*^2^** diff = chi square difference; Pr(>Chisq) = p-value for the chi square test, tests compare consecutive models; **AIC** = Akaike information criterion; **CFI** = comparative fit index; **TLI** = Tucker–Lewis index; **RMSEA** = root mean square error of approximation; 95%CI RMSEA = 95% confidence intervals for RMSEA; **Path** = step change for each model. All results are averages and 95%CI intervals from 1000 iteration boot strap estimates of model fit.

**Table 3:** Structural equation regressions, total and indirect effects of H8 model

Regressions	Estimate	Standard Errors	z-value	P(>|z|)	Std.all
*Case by* ~					
Obsessionality	0.03	0.005	5.15	<0.001	0.26
Sensation seeking	0.01	0.005	2.01	0.04	0.08
PUI	0.01	0.003	3.34	0.001	0.15
*PUI by* ~					
Obsessionality	0.463	0.074	6.23	<0.001	0.30
Sensation seeking	0.275	0.069	3.97	<0.001	0.16
**Total effects**					
Obsessionality on case	0.032	0.005	6.23	<0.001	0.30
Sensation seeking on case	0.012	0.005	2.59	0.01	0.11
**Indirect effects**					
PUI~obsessionality	0.005	0.002	3.02	0.002	0.044
PUI~sens.seeking	0.003	0.001	2.57	0.01	0.024

**Legend: SS** = sensation seeking latent factor; **Obs** = obsessionality latent factor; **OBQ** = Obsessive Beliefs Questionnaire, short version (OBQ-20); **OBQ m** = Obsessional thoughts of importance and control; **OBQ n** = Obsessional intolerance towards uncertainty; **OBQ p** = Obsessional thoughts of inflated responsibility; **OBQ t** = Obsessional thoughts of threat; **Problematic Usage of the Internet (PUI)** = Internet Addiction Test, short version (IAT-12) total score; **z-values** = regression coefficients; **Std.all** = standardized coefficients; **Case** = SCOFF > 2, Exercise Addiction Inventory > 18.
